# The Novel HLA‐E*01:152 Allele Identified in African Populations Using Next‐Generation Sequencing

**DOI:** 10.1111/tan.70247

**Published:** 2025-05-13

**Authors:** Nitalia Naidoo, Tiza L. Ng'uni, Uvedhna Padia, Alabi W. Banjoko, Zaza M. Ndhlovu

**Affiliations:** ^1^ Africa Health Research Institute (AHRI), Nelson R. Mandela School of Medicine Durban South Africa; ^2^ Department of Statistics University of Ilorin Kwara Nigeria; ^3^ Ragon Institute of Massachusetts General Hospital, Massachusetts Institute of Technology, and Harvard University Cambridge Massachusetts USA

**Keywords:** HLA‐E*01:152, next‐generation sequencing, novel allele, variant

## Abstract

E*01:152 differs from E*01:03:05:01 by a non‐synonymous A>G substitution at gDNA position 958 (exon 3).

## 1 Communication

We report a novel HLA‐E allele, now named HLA‐E*01:152, identified during high‐resolution HLA genotyping of African populations using Next‐Generation Sequencing (NGS). This variant was detected in 12 individuals across four countries—six in Zimbabwe, four in South Africa, one in Zambia, and one in Kenya—prompting further investigation.

Genomic DNA was extracted from peripheral blood mononuclear cells (PBMCs) using the Qiagen QIAamp DNA Midi Kit (Qiagen, Hilden, Germany). DNA concentration averaged 280.11 ng/μL with an optical density (OD) 260/280 ratio of 1.82. Amplification of the classical HLA loci and HLA‐E was performed by polymerase chain reaction using the GenDx NGSgo‐MX11‐3 and NGSgo‐AmpX v2 kits, respectively. Library preparation included DNA fragmentation, adapter ligation, dual indexing, and magnetic bead purification using the GenDx NGSgo Library Full Kit (GenDx, Utrecht, Netherlands). Following fragment analysis and quantification of the final libraries, sequencing was performed on the Illumina NextSeq 2000 platform with paired‐end 150 bp reads using P1 XLEAP‐SBS reagent kits (Illumina, San Diego, California). Data analysis using GenDx NGSengine (v3.1.2.33659) identified unique patterns not defined in IPD‐IMGT/HLA Database 3.55.0 [1], suggesting the presence of a novel HLA‐E variant (informed consent was obtained from all participants prior to their enrolment in the study).

**FIGURE 1:**
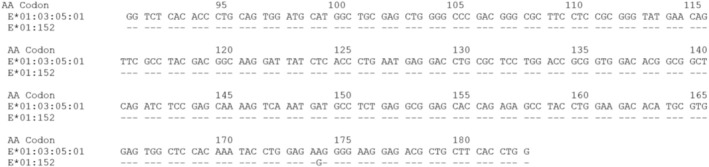
Alignment of the sequence of exon 3 of E*01:152 with the sequence of E*01:03:05:01. Dashes indicate nucleotide identity with E*01:03:05:01 (reference allele). Numbers above the sequence indicate codon position. In the E*01:152 allele, the identified substitution occurs as a change from A>G in codon 174.

E*01:152 differs from E*01:03:05:01 by a single A>G substitution at genomic position 958 in exon 3. This nucleotide change results in a missense mutation in codon 174 (AAG>AGG), changing residue 174 Lysine to Arginine within the α2 domain (Figure 1).

The E*01:152 allele frequencies were 1.36% in Zimbabwe, 1.02% in South Africa, 0.75% in Zambia and 0.52% in Kenya. Interestingly, a strong association between E*01:152 and A*29:11 was observed. Among the 580 individuals genotyped, 10 carried E*01:152 and A*29:11, 2 carried only E*01:152, 1 carried A*29:11 only, and 567 had neither allele. Fisher's exact test showed that individuals with E*01:152 are approximately 2835 times more likely to carry A*29:11 compared to those without E*01:152 (OR: 2835, 95% CI = [249.1, 30012], *p* < 0.0001). Statistical analyses were performed using GraphPad Prism v10.0.3.

These findings suggest a strong linkage disequilibrium and co‐inheritance. The possibility that the A*29:11 leader peptide preferentially stabilises E*01:152 on the cell surface warrants further investigation. Notably, the codon 174 Lysine to Arginine substitution in E*01:152 is located in the HLA‐E heavy chain's α2 domain, which, together with the α1 domain, forms the peptide‐binding groove for peptide presentation to immune cells [2]. Replacing Lysine with Arginine introduces a larger guanidinium group in place of Lysine's ε‐amino group, potentially altering local folding or electrostatic interactions. Furthermore, Arginine's delocalised positive charge may affect peptide conformation or receptor binding [3]. These biochemical changes may impact the repertoire of peptides presented by HLA‐E, its surface expression, and its recognition by CD94/NKG2A on NK cells or CD8+ T cells, thereby modulating immune responses [4]. Structural modelling with functional assays and association studies is necessary to elucidate the evolutionary significance of this substitution and its potential impact on disease susceptibility.

The sequence of E*01:152 has been submitted to the GenBank nucleotide sequence database under accession number PQ587575. The name E*01:152 has been officially assigned by the WHO Nomenclature Committee for Factors of the HLA System in January 2025. This follows the agreed policy that, subject to the conditions stated in the most recent Nomenclature Report [5], names will be assigned to new sequences as they are identified. Lists of such new names will be published in the following WHO Nomenclature Report.

## Conflicts of Interest

The authors declare no conflicts of interest.

## Data Availability

The data that support the findings of this study are openly available in IPD‐IMGT/HLA at https://www.ebi.ac.uk/ipd/imgt/hla/alleles/allele/?accession=HLA42368, reference number PQ587575.

## References

[1] J. Robinson , D. J. Barker , and S. G. E. Marsh , “25 Years of the IPD‐IMGT/HLA Database,” HLA 103, no. 6 (2024): e15549. 38936817 10.1111/tan.15549

[2] C. A. O'Callaghan and J. I. Bell , “Structure and Function of the Human MHC Class Ib Molecules HLA‐E, HLA‐F and HLA‐G,” Immunological Reviews 163 (1998): 129–138. 9700506 10.1111/j.1600-065x.1998.tb01192.x

[3] M. J. Betts and R. B. Russell , “Amino Acid Properties and Consequences of Substitutions,” in Bioinformatics for Geneticists, ed. M. R. Barnes , and I. C. Gray (Wiley, 2003), 289–316.

[4] R. K. Strong , M. A. Holmes , P. Li , L. Braun , N. Lee , and D. E. Geraghty , “HLA‐E Allelic Variants. Correlating Differential Expression, Peptide Affinities, Crystal Structures, and Thermal Stabilities,” Journal of Biological Chemistry 278, no. 7 (2003): 5082–5090. 12411439 10.1074/jbc.M208268200

[5] S. G. Marsh , E. D. Albert , W. F. Bodmer , et al., “Nomenclature for Factors of the HLA System,” Tissue Antigens 75, no. 4 (2010): 291–455. 20356336 10.1111/j.1399-0039.2010.01466.x PMC2848993

